# Nutrition and Social Disadvantage as Risk Factors for Mortality Among School-Age Children: Regional Differences in Kazakhstan

**DOI:** 10.3390/ijerph23010039

**Published:** 2025-12-27

**Authors:** Zulfiya Yelzhanova, Jainakbayev Nurlan, Madina Kamalieva, Karlygash Zhubanysheva, Anna Tursun

**Affiliations:** 1 Department of Public Health, Non-Profit Educational Institution “Kazakhstan-Russian Medical University”, Almaty 050004, Kazakhstan; zulfiy986@gmail.com (Z.Y.); rector@medkrmu.kz (J.N.); kamalieva.m@medkrmu.kz (M.K.); 2 Department of Neonatology, Non-Profit Educational Institution “Kazakhstan-Russian Medical University”, Almaty 050004, Kazakhstan; k.zhubanysheva@medkrmu.kz

**Keywords:** school-age child mortality, Kazakhstan, regional differences, causes of death (ICD-10), nutrition, socio-economic factors, ecological analysis, linear mixed-effects regression, health inequality

## Abstract

Objective: To assess the structure and regional variation in mortality among school-aged children in Kazakhstan from 2015 to 2024, and to determine its association with dietary patterns and socio-economic factors. Materials and Methods: An ecological inter-regional analysis was conducted using official statistical data of the Republic of Kazakhstan. Mortality rates among children aged 6–17 years, the distribution of death causes according to ICD-10, indicators of consumption of major food product groups, and poverty levels were examined. Linear mixed-effects regression with a random intercept for region and fixed effects for year and covariates, and spatial description of regional trends were applied. Results: Variation in school-age mortality across regions and calendar years was evident, with external causes predominating, followed by diseases of the nervous system, neoplasms, and diseases of the circulatory and respiratory systems in the mortality structure. In the multivariable linear mixed-effects model, none of the dietary or socioeconomic predictors showed statistically significant independent associations with mortality (all *p* > 0.05), and the calendar year was not significant (*p* = 0.180). Model explanatory power was very low (marginal R^2^ = 0.017; conditional R^2^ = 0.020; ICC = 0.005), and residuals demonstrated significant temporal autocorrelation (*p* < 0.001). Conclusions: The mortality structure among school-aged children is shaped by a complex interplay of medical, social, and behavioral determinants. Dietary and socioeconomic indicators showed only weak ecological associations with mortality and did not retain independent effects after multivariable adjustment, underscoring the multifactorial nature of regional mortality patterns and the need for multisectoral action, including improved access to nutritious foods, enhanced social well-being, and strengthened health system capacity.

## 1. Introduction

The right to health is recognized as one of the fundamental human rights. Universal Health Coverage (UHC) is a key mechanism for the realization of this right and constitutes an integral component of the United Nations 2030 Agenda. Within the Sustainable Development Goals (SDGs), UHC is reflected in Goal 3 “Good Health and Well-Being” (target 3.8) [1].

In 2019, mortality among children and adolescents aged 5–19 years was estimated at approximately 1.48 million globally, according to Li Liu et al. The leading causes of death in this age group included road traffic injuries, cancer, malaria, drowning, and diarrheal diseases. The authors concluded that the main causes of death among school-aged children are largely preventable [2].

International agencies such as WHO, UNICEF, and IHME do not routinely publish mortality statistics specifically for the 6–17-year age group, as it does not correspond to the standard global reporting categories (5–9, 10–14, 15–19 years) [2,3,4]. As a result, there are no publicly available international datasets providing global or regional mortality estimates disaggregated exclusively for children aged 6–17 years.

A rare example addressing mortality specifically in school-aged children is the 2021 study “Sudden Death of School-Aged Children During Physical Activity,” which analyzed deaths in the 8–17-year age range. The authors showed that most fatal outcomes were associated with previously undiagnosed cardiac conditions [5].

Although cardiomyopathies in children and adolescents are predominantly genetic or congenital, recent evidence underscores the important role of nutrition as a modifiable factor [6], particularly deficiencies in micronutrients such as selenium [7,8]. Selenium is one of the key trace elements for cardiovascular function, thyroid hormone metabolism, antioxidant defense, and both adaptive and innate immunity [9]. The daily requirement for selenium in children is 30–50 μg, and main dietary sources include marine fish and seafood (tuna, sardines, herring, cod, shrimp, etc.), liver, eggs, milk and dairy products, poultry, grains and cereals, nuts, garlic, and onions [10].

Thus, nutrition plays a crucial role in maintaining physiological cardiovascular function in school-aged children. Problems of undernutrition and unbalanced diets are therefore of particular concern. The case report “Malnutrition-Associated Cardiomyopathy in a Child With Autism Spectrum Disorder” [11] provides additional evidence of the critical role of adequate nutrition in preserving normal cardiovascular function in children.

Socioeconomic factors are among the key determinants of child mortality [12,13]. Poverty—typically defined as the proportion of the population with incomes below the subsistence minimum—is associated with an increased risk of child mortality, including among school-aged children [2,4,14].

Poverty remains a pressing issue in the Republic of Kazakhstan and directly affects the quality of life and population health. In 2022, 5.1% of the population—around 500,000 people—were living below the poverty line [13].

A meta-analysis published in 2025, which reviewed 224 studies worldwide indexed in international databases (44.6% of all analyzed publications), found that 100 studies demonstrated an association between socioeconomic status and child mortality. The probability of death among children under five years of age in countries with high levels of poverty was five times higher, while in school-aged groups, mortality risk was 1.5–2 times higher [15].

In addition, undernutrition and unbalanced diets are associated with a high risk of mortality in children [16]. Imbalances in dietary structure accompanied by deficiencies of essential nutrients contribute to the development of chronic nutrition-related diseases, including gastrointestinal disorders, metabolic disturbances, endocrine diseases, and cardiovascular conditions [17,18,19].

The relationship between school-age mortality, nutritional indicators, and socioeconomic status is grounded in the concept of social and medical determinants of health. According to this framework, diet quality, access to healthy foods, income level, and living conditions together shape key risks for child morbidity and mortality [20,21,22]. Nutrition reflects both biological and social well-being: insufficient consumption of dairy products, vegetables, and fruits reduces immune function, increases susceptibility to infections, and adversely affects cognitive development [23,24]. Poverty limits access to a diverse diet, regular medical care, and preventive services, thereby reinforcing health inequalities among children [25,26,27]. Consequently, an integrated analysis of mortality, nutrition, and poverty makes it possible to more objectively assess the combined determinants of risk, which cannot be adequately interpreted in isolation [28]. People living in poverty often lack the amount and quality of food required to obtain essential nutrients [28]; this problem is particularly critical for children, as studies show that malnourished children from poor households have a higher risk of illness and premature death [29].

In Kazakhstan, there is a shortage of studies that provide a comprehensive analysis of mortality among school-aged children. Regional heterogeneity, socioeconomic determinants, nutritional factors, and access to medical care have been examined only fragmentarily.

The present study is aimed at addressing this research gap and represents the first multi-year, nationwide assessment of causes and determinants of school-age mortality in Kazakhstan. The work is carried out within the framework of program-targeted funding from the Ministry of Health of the Republic of Kazakhstan (Project No. BR27310319 “Development of preventive and rehabilitation programs to improve population quality of life in the post-COVID period”), which emphasizes its relevance for the national health system.

The objective of this study was to identify regional differences in mortality among school-aged children in Kazakhstan over the period 2015–2024 and to assess their association with dietary and socioeconomic factors, including consumption of major food groups and poverty levels.

## 2. Materials and Methods

### 2.1. Study Design and Data Sources

This study was conducted as an ecological study based on panel aggregated “region–year” data for the Republic of Kazakhstan for the period from 1 January 2015 to 31 December 2024.

The analytical sample included 20 administrative regions (17 oblasts and 3 cities of republican significance) and up to 10 calendar years of observation per region, yielding a total of 2950 region–year observations for mortality indicators and 2647 and 2663 observations for the full set of socioeconomic and dietary indicators (the actual number depending on data availability for specific variables).

Data on mortality among children aged 6–17 years were provided by the Salidat Kairbekova National Scientific Center for Health Development as an official cleaned and standardized dataset. All national statistical data undergo mandatory procedures of validation and quality control. Mortality indicators for children aged 6–17 years were presented as crude age-specific mortality rates per 100,000 children in the corresponding age group. No recalculation of the original mortality rates was performed.

### 2.2. Socioeconomic and Dietary Indicators

#### 2.2.1. Consumption of Major Food Groups

Data on the consumption of major food groups were obtained from publicly available statistical tables of the Bureau of National Statistics of the Agency for Strategic Planning and Reforms of the Republic of Kazakhstan.

All indicators reflect average annual per capita consumption at the regional level and are not specific to the 6–17-year age group. They characterize the mean level of consumption in the general population of the region and, within the ecological study design, are used as proxy measures of the regional food environment.

Units of measurement were as follows:

- kg per capita per year—bread and cereals, meat and meat products, fish and seafood, oils and fats, vegetables, fruits, potatoes, sugar and confectionery;
- liters per capita per year—milk and dairy products;
- pieces per capita per year—eggs.

All indicators were aggregated by calendar year and region and were linearly matched to the corresponding annual regional mortality rates for children aged 6–17 years.

#### 2.2.2. Poverty Indicators

Poverty level was characterized by the proportion of the population with incomes below the subsistence minimum, expressed as a percentage of the total regional population. The analysis also included the indicator “Share of the population with incomes below the cost of the food basket,” which reflects the extent of low income. This indicator is defined as the ratio of the number of individuals whose incomes do not reach the cost of the food basket to the total population, expressed in percentage terms.

The indicator has been calculated by the Committee on Statistics since 2001 based on household budget surveys, including daily expenditure diaries (form D003), quarterly questionnaires on the structure of income and expenditure (form D004), and household composition control cards (form D008).

Both indicators were aggregated at the “region–year” level and used to characterize the macroeconomic context; they do not capture the individual socioeconomic status of the families of deceased children.

#### 2.2.3. Gross Regional Product (GRP)

To characterize the macroeconomic level, we used gross regional product (GRP), defined as the market value of all final goods and services produced by regional residents over one year. GRP was expressed in million tenge and calculated using the production method according to the official methodology of the Bureau of National Statistics.

### 2.3. Handling of Missing Data

In all data sources, missing values were coded as NA rather than as zeros, in order to avoid artificial underestimation of mean values and bias of regression estimates.

Missing values for the newly established administrative regions (Abai, Zhetysu, Ulytau, Atyrau and the city of Shymkent in periods prior to their formation) were also kept as NA and were not included in the calculation of aggregated indicators.

In the descriptive statistics, the number of available observations (n) was explicitly reported for each variable. In correlation and regression analyses, a complete case analysis approach was used: each model included only observations with full data on the relevant variables.

There were no missing values for key mortality indicators; for socioeconomic and dietary indicators, the proportion of missing observations did not exceed 11%.

### 2.4. Regression Analysis

To assess associations between regional mortality rates among children aged 6–17 years and socioeconomic and dietary characteristics, we applied a linear mixed-effects regression model with a random intercept for region.

Fixed effects:

− per capita consumption of major food groups;
− proportion of the population with incomes below the subsistence minimum;
− gross regional product;
− calendar year of observation.

Random effect: region (random intercept).

All analyses were conducted using a complete case analysis approach.

Multicollinearity among predictors was evaluated using variance inflation factors (VIF).

Model performance was assessed using:

− marginal R^2^—variance explained by fixed effects;
− conditional R^2^—variance explained by fixed and random effects combined;
− intraclass correlation coefficient (ICC)—proportion of between-region variance.

Temporal autocorrelation of residuals was examined using statistical tests and graphical diagnostics. Interpretation explicitly accounted for the panel nature of the data and potential nonlinearity over time.

### 2.5. Statistical Analysis

Statistical analyses were performed in R version 4.5.2 (R Foundation for Statistical Computing, Vienna, Austria). The following packages were used for data preparation and analysis: dplyr for data selection, filtering, and aggregation; tidyr for constructing the “region–year” panel structure; DescTools for descriptive statistics, including medians and interquartile ranges; ggplot2 for plotting temporal trends and faceted panels; stringr for processing text variables and harmonizing ICD-10 codes; forcats for ordering and ranking categorical variables (regions); and, where necessary, car, lmtest, nlme, and lme4 for diagnostic testing and sensitivity analyses of regression models.

## 3. Results

**Figure 1:**
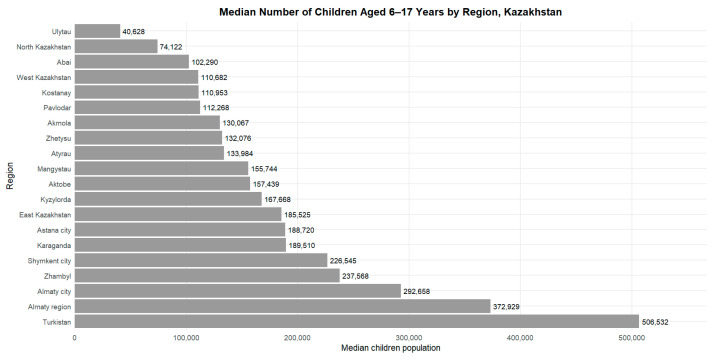
Median number of children aged 6–17 years by region of Kazakhstan, 2015–2024.

Figure 1 and Table 1 present the number of children aged 6–17 years across the regions of Kazakhstan for the period 2015–2024. The median number of school-aged children ranged from 40,628 in the Ulytau region to 506,532 in the Turkistan region (Table 1). High values were also observed in the cities of republican significance (Almaty, Astana and Shymkent), where the median number of children exceeded 180,000. In the remaining regions, the median values ranged from 74,000 to 238,000 children.

Over the period 2015–2024, the median number of children aged 6–17 years across the regions of Kazakhstan ranged from 40,628 (95% range: 39,481–41,218) in the Ulytau region to 506,532 (469,110–521,723) in the Turkistan region (Table 1). High values were also observed in the cities of republican significance: Almaty—292,658 (253,952–331,436), Shymkent—226,545 (200,275–251,596), and Astana—188,720 (148,959–238,189).

The lowest values were recorded in North Kazakhstan region—74,122 (72,622–75,171), West Kazakhstan region—110,682 (100,198–119,580), and Kostanay region—110,953 (105,897–114,715).

**Figure 2:**
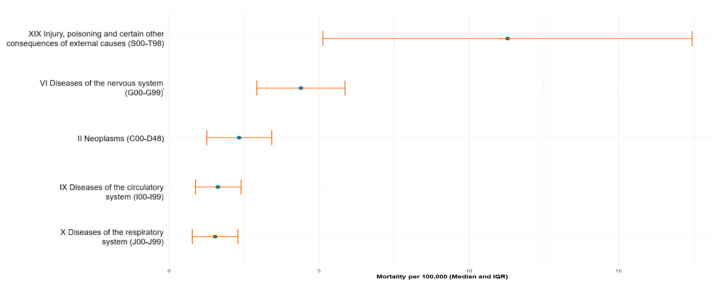
Top-5 leading causes of mortality among children aged 617 years in Kazakhstan from 2015 to 2024. Data are presented as median mortality rate per 100,000 population with interquartile range (IQR), calculated based on official national statistics.

In the 6–17-year age group, external causes (ICD-10: S00–T98) were the major contributors to mortality, with a median age-specific mortality rate of 11.29 deaths per 100,000 children (IQR 5.13–17.45). These were followed by diseases of the nervous system (G00–G99)—4.39 (2.92–5.87), neoplasms (C00–D48)—2.34 (1.26–3.42), diseases of the circulatory system (I00–I99)—1.63 (0.87–2.40), and respiratory diseases (J00–J99)—1.53 (0.77–2.29) (Table 2, Figure 2).

**Figure 3:**
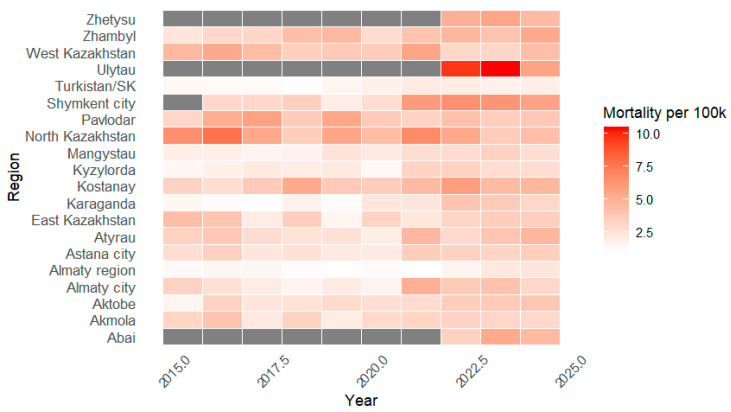
Annual mortality rates among children aged 6–17 years (per 100,000) by region of Kazakhstan, 2015–2024. Periods without official data for the newly established administrative regions are shown in gray.

Figure 3 presents the trends in age-specific mortality among children aged 6–17 years across the regions of the Republic of Kazakhstan in 2015–2024. In most regions, mortality rates fluctuated between 1 and 5 deaths per 100,000 children, with marked variation between territories and calendar years. The highest values were recorded in North Kazakhstan region in several years prior to 2022, as well as in Ulytau region in 2022–2023. Turkistan, Almaty region and Karaganda region consistently demonstrated the lowest mortality levels over the entire observation period. For three regions (Abai, Ulytau and Zhetysu), data are available only from 2022 onwards due to administrative reorganization.

**Figure 4:**
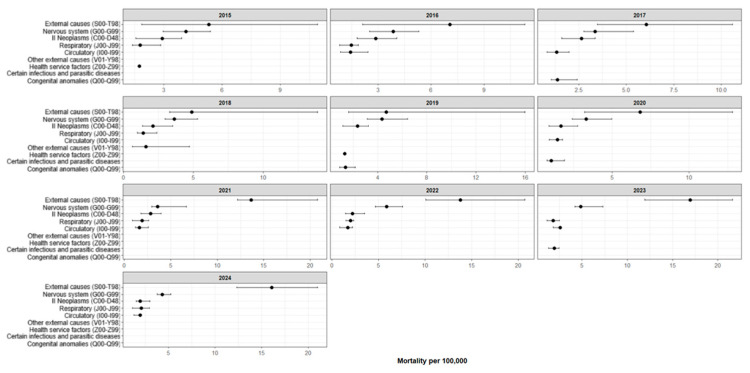
Five leading causes of death among children aged 6–17 years in Kazakhstan by year (median per 100,000 population with IQR), 2015–2024. Compiled by the authors.

Figure 4 presents the five most frequent causes of death among children aged 6–17 years in the Republic of Kazakhstan by year of observation (2015–2024). In all study years, the leading cause of death was the category “Injury, poisoning and certain other consequences of external causes” (ICD-10: S00–T98), with median age-specific mortality rates ranging from 4.69 to 16.96 per 100,000 population. The second most common cause was consistently “Diseases of the nervous system” (ICD-10: G00–G99), with median mortality ranging from 3.30 to 5.87 per 100,000. “Neoplasms” (ICD-10: C00–D48) typically ranked third, with median levels of 1.67–2.91 per 100,000. For “Diseases of the respiratory system” (ICD-10: J00–J99), rates remained within 1.42–2.10 per 100,000.

The fifth position was occupied either by “Diseases of the circulatory system” (ICD-10: I00–I99; 1.34–2.58 per 100,000) or, in some years, by “Congenital malformations, deformations and chromosomal abnormalities” (ICD-10: Q00–Q99) and “Certain infectious and parasitic diseases” (ICD-10: A00–B99). Overall, the pattern of leading causes of death remained relatively stable throughout the study period.

**Figure 5:**
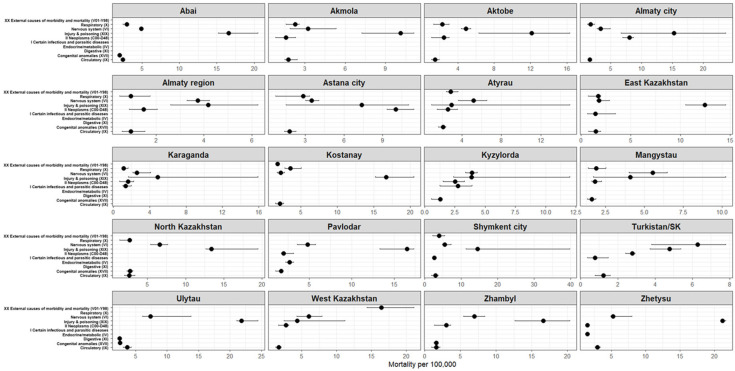
Top five causes of death among children aged 6–17 years by region of the Republic of Kazakhstan (median per 100,000 population with IQR), 2015–2024. Compiled by the authors.

Figure 5 presents the five most common causes of death among children aged 6–17 years across the regions of Kazakhstan for the period 2015–2024 (Complete region-by-cause estimates for the top five causes are provided in Supplementary Table S1). In all regions, the largest share of mortality is accounted for by “Injury, poisoning and certain other consequences of external causes” (ICD-10: S00–T98), although the magnitude of the rates varies substantially by territory.

The highest median mortality rates from external causes were observed in Ulytau (21.84 [21.05–24.46] per 100,000), Zhetysu (21.20 [21.10–21.67]), Zhambyl (16.65 [12.63–20.34]), Pavlodar (16.53 [13.30–17.34]), Kostanay (16.67 [15.23–20.47]), Shymkent city (14.48 [11.32–39.75]) and East Kazakhstan (12.46 [10.53–14.56]).

In most regions, the second leading group of causes is “Diseases of the nervous system” (ICD-10: G00–G99), with particularly high levels in Ulytau (7.38 [6.12–13.82]), Zhambyl (6.95 [5.46–8.43]), West Kazakhstan (6.01 [4.31–7.94]), North Kazakhstan (6.64 [5.39–7.65]) and Mangystau (5.54 [4.02–6.47]).

In the majority of regions, “Neoplasms” (ICD-10: C00–D48) rank third, with rates exceeding 2.5 per 100,000 in Almaty city, Astana city, Aktobe, Pavlodar, Kostanay, Kyzylorda and Turkistan/SK.

Other causes that enter the top five depending on the region include

- “Diseases of the respiratory system” (ICD-10: J00–J99);
- “Diseases of the circulatory system” (ICD-10: I00–I99);
- “Congenital malformations, deformations and chromosomal abnormalities” (ICD-10: Q00–Q99);
- “Certain infectious and parasitic diseases” (ICD-10: A00–B99);
- “Endocrine, nutritional and metabolic diseases” (ICD-10: E00–E90);
- “Symptoms, signs and abnormal clinical and laboratory findings, not elsewhere classified” (ICD-10: R00–R99).

Despite regional differences in absolute mortality levels, the overall pattern of leading causes of death among school-aged children was broadly comparable across regions.

Table 3 presents the median per capita consumption of major food products and gross regional product (GRP) by region of the Republic of Kazakhstan over the study period.

Per capita food consumption varied substantially between regions. The highest median intake of bread and bakery products was observed in the Turkistan region—172.3 (164.9–173.3) kg per capita per year—while the lowest was recorded in Almaty city—103.8 (103.4–110.8) kg.

Meat consumption was highest in the Almaty region and East Kazakhstan region—93.4 (93.2–97.2) kg and 87.8 (86.4–90.3) kg, respectively—whereas Shymkent city showed the lowest level at 54.5 (53.1–58.2) kg.

Fish consumption ranged from 5.4 (4.9–8.8) kg in Shymkent city to 20.2 (15.6–21.2) kg in North Kazakhstan region. Milk consumption also differed markedly: the highest median level was observed in East Kazakhstan region—289.1 (276.1–294.9) L per capita per year—and the lowest in Shymkent city—165.7 (155.7–208.0) L.

The highest consumption of vegetables and fruits was recorded in Almaty city: 97.6 (95.4–99.4) kg and 90.8 (78.9–97.8) kg per capita, respectively. Potato consumption ranged from 29.6 (28.7–30.7) kg in the Abai region to 59.9 (50.7–60.8) kg in the Karaganda region.

Median GRP per capita also showed pronounced regional differences: the lowest values were observed in Zhetysu region—1,618,150 (1,376,926–1,914,049) tenge per capita—and the highest in Almaty city—13,503,381 (11,893,226–19,154,537) tenge per capita.

Table 4 presents the proportion of the population with incomes below the subsistence minimum by region. The lowest poverty levels were observed in Pavlodar region—0.0% (0.0–0.0)—as well as in Karaganda, Kostanay and Almaty regions and in the metropolitan cities, where the median did not exceed 0.2%.

The highest poverty level was recorded in Ulytau—0.6% (0.5–0.6)—while in Shymkent the median reached 0.3% (0.1–0.7). No data were available for the Atyrau region. Overall, poverty indicators remained low across all regions of Kazakhstan during the study period.

The results of the multivariable linear mixed-effects modeling are presented in Table 5. None of the dietary or socioeconomic predictors showed a statistically significant association with mortality among children aged 6–17 years after adjustment for all covariates (all *p* > 0.05).

The calendar year of observation was not a significant independent predictor (*p* = 0.180), indicating no evidence of a linear time trend in the model. The marginal coefficient of determination was low (R^2^m = 1.7%), and the conditional R^2^ was similarly small (R^2^c = 2.0%), suggesting that the included predictors explained only a very limited proportion of the variance in mortality rates.

The intraclass correlation coefficient (ICC) was 0.5%, indicating that between-region differences accounted for less than 1% of the total variance, i.e., geographical heterogeneity was minimal. Residual diagnostics revealed significant autocorrelation (*p* < 0.001), indicating temporal dependence of observations within regions and underscoring the panel structure of the data.

Assessment of multicollinearity indicated that all predictors were within acceptable limits (all VIF < 4). Moderate collinearity was observed for consumption of meat, dairy products, eggs, fruits and the “year” variable, which is expected given the shared directionality of dietary indicators and the presence of an overall time trend. Poverty level showed very low collinearity (VIF = 1.19), allowing its effect to be interpreted as relatively independent.

Analysis of residual autocorrelation revealed statistically significant temporal dependence of observations within regions (*p* < 0.001), confirming the panel structure of the data and the interdependence of consecutive measurements over 2015–2024.

The contribution of the random effect “region” was minimal: the adjusted ICC was 0.005, meaning that less than 1% of the variance in mortality was explained by between-region differences. Thus, most of the variability was driven by temporal changes, while geographical heterogeneity was very limited.

Model diagnostics showed marked heteroscedasticity, heavy right tails on the Q–Q plot and several outlying observations, predominantly among those with high predicted values. These features indicate violations of standard linear model assumptions and suggest potential non-linearity in the temporal dynamics.

Taken together with the detected autocorrelation, the low explanatory power of the model (marginal R^2^ = 1.7%) and the minimal contribution of between-region differences, these findings point to only a weak ecological relationship between mortality and indicators of diet, poverty and economic development. This underscores the need for alternative analytical approaches that better account for temporal dependence and the non-standard distribution of the outcome variable.

## 4. Discussion

The difference in age ranges is driven by the fact that Kazakhstan uses an education-based principle for defining age groups [30], whereas international agencies (WHO, UNICEF, UN IGME) apply a demographic approach. In Kazakhstan, children typically enter school at age 6 and complete it at age 17; therefore, the 6–17-year range reflects the national definition of school age. By contrast, international institutions use standardized age intervals (5–9, 10–14, 15–19 years), which facilitate cross-country comparisons in settings with different ages at school entry [31,32].

The 6–17-year range is therefore not used globally, as it is anchored in the national education system rather than in international demographic conventions.

### 4.1. Role of Diet and Food Security

The impact of diet and socioeconomic conditions on child mortality remains multifactorial. Despite the decline in global mortality among younger children, a substantial proportion of premature deaths still occurs among school-aged children. According to the WHO, in 2020, there were approximately 869,000 deaths among children aged 5–14 years, and the probability of dying between ages 5 and 14 remained around 7 per 1000 children aged 5 years [33]. UNICEF reports that in 2023, there were about 2.1 million deaths among children and young people aged 5–24 years, underscoring the vulnerability of adolescents [32].

Undernutrition and poor dietary quality are key modifiable determinants of child morbidity and mortality. Undernutrition (stunting, wasting, micronutrient deficiencies) increases the risk of infectious diseases [34]. Among school-aged children, both undernutrition and excess body weight are common. In 2022, almost one in five children and adolescents aged 5–19 years was overweight, and obesity affected 8%, a figure markedly higher than in 2000 [35].

The review by Choedon et al. on South Asian countries demonstrates wide variability in the prevalence of undernutrition among schoolchildren: wasting ranged from 3% to 48%, and stunting from 3.7% to 71.7% [36]. Some studies also report high proportions of children who are overweight. Russian data indicate that a preference for sugary drinks and frequent consumption of sweets is typical for most schoolchildren, reflecting an energy-dense but nutritionally poor diet [37].

Country-specific data help to clarify the role of food security. In an Indonesian study, the risk of stunting was higher among children from households at risk of food insecurity; energy deficiency also significantly increased the likelihood of stunting [38]. Food insecurity amplifies wasting and underweight, especially when combined with poor sanitation [39,40].

According to the Bureau of National Statistics (2024), households in Kazakhstan allocate a substantial share of their expenditures to food. Meat and meat products account for 16.9% of total spending, while bread and cereals account for 7.7% [41]. For comparison, in European Union countries the average share of food expenditures is 14.3%. In Eastern Europe these values tend to be higher than in Western Europe (e.g., Ireland—8.3%, Luxembourg—9.0%). These differences correlate with variation in health indicators and dietary patterns across European regions [42,43].

Taken together, differences in the availability and structure of diets translate into variability in children’s anthropometric indicators and infection risk. However, the strength of these associations is strongly influenced by sanitation, coverage of health services and the broader socioeconomic context.

### 4.2. Poverty as a Social Determinant

Poverty remains a key social determinant of mortality among school-aged children. WHO estimates indicate that most of the roughly 1 million deaths in the 5–14-year age group in 2021 occur in low-income countries. Global Burden of Disease (GBD) 2019 analyses show that mortality among 5–19-year-olds in low-SDI countries is 5–12 times higher than in high-SDI settings [4]. UNICEF also reports that adolescents from the poorest households have a substantially higher risk of death. These findings highlight the importance of access to healthcare, sanitation and education [32].

### 4.3. Regional Disparities in Child Mortality Within Countries

Regional differences in mortality among school-aged children remain substantial even in countries with a formally uniform national income level. WHO data show that deaths among 5–14-year-olds are predominantly concentrated in areas with weak health infrastructure and limited access to basic services (“most deaths among 5–14-year-olds occur in low-income settings”, WHO) [33]. In sub-Saharan Africa, spatial analyses have identified persistent “hotspots” of elevated mortality that coincide with zones of low coverage by health services, poor sanitation and large distances to health facilities [14,44,45].

GBD 2019 also reveals marked within-country variation: mortality among 5–19-year-olds is substantially higher in regions with a low sociodemographic index (SDI), even when more developed areas exist within the same country [46]. These intra-country disparities reflect the impact of poverty, rural residence, limited access to qualified care and shortcomings in safety-related infrastructure. Overall, the data confirm that regional heterogeneity remains a key driver of mortality among school-aged children and is shaped by a combination of medical, sanitary and socioeconomic conditions [2].

Studies that simultaneously assess dietary and socioeconomic factors are of particular interest. Ogunniyi et al., in an analysis of 11,655 households in Nigeria, showed that a transition from food insecurity to food security was associated with an approximately one-quarter reduction in the probability of child death, with a stronger effect in urban (≈26%) than in rural (≈11%) areas [47]. Maternal education, access to healthcare and belonging to specific geopolitical zones also had significant effects.

### 4.4. Comparison with the Findings of the Present Study

Our results indicate that the relationships between dietary patterns, poverty and mortality among school-aged children are multidimensional and cannot be captured by a simple linear framework. Correlation analysis showed weak or moderate associations: the positive correlation between fruit consumption and mortality (ρ = 0.227) suggests the presence of a relationship, but its magnitude explains only a small portion of inter-regional variation. Similarly, correlations with other food groups were low, which is consistent with international evidence showing that dietary factors constitute only one component of a complex set of determinants linked to sanitation, access to healthcare and social context [20,22,25,28].

The moderate positive correlation between consumption of meat and dairy products (ρ = 0.526) reflects dietary patterns in regions with higher prosperity and better access to more expensive foods. In the literature, such patterns are often interpreted as markers of higher socioeconomic status and “westernization” of the diet, which may have both beneficial effects (better protein and calcium intake) and adverse consequences (increased risk of non-communicable diseases) [17,43].

The near-zero correlation between regional poverty level and mortality (ρ = 0.028) requires careful interpretation. Several explanations are possible:

The effect of poverty may be attenuated against the background of relatively high baseline levels of medical and sanitary services in an upper-middle-income country, where income differences contribute less to mortality variation [15,20,25].

The regional poverty indicator is relatively crude and does not capture within-region inequalities. The most vulnerable households may be concentrated in specific neighborhoods, without substantially affecting the regional average [12,14,15,27].

Features of death registration and cause-of-death structure may play a role: as infectious mortality declines, the relative contribution of perinatal conditions, congenital anomalies and injuries increases, and these may be more prominent in economically developed regions with better diagnostic capacity [2,3,46].

Taken together, our findings are consistent with current literature: at an aggregated level, direct associations between diet, poverty and mortality are weak. However, at the individual level, food security, dietary quality, access to preventive services and the family’s socioeconomic status form a complex network of interrelated factors that determine risks of stunting and death [23,24,28,39,40,44,45]. This underscores the need for comprehensive multilevel models that take into account both inter-regional differences and within-region inequalities.

### 4.5. Limitations

This study is subject to the risk of ecological fallacy, as associations identified at the aggregated level do not necessarily reflect individual-level relationships [48,49,50]. Regional indicators of food consumption do not directly correspond to the diets of school-aged children and more likely characterize household consumption patterns as a whole. In addition, the 2015–2024 data may be affected by measurement errors, methodological changes and the consequences of the COVID-19 pandemic. Therefore, the findings should be interpreted as descriptive and hypothesis-generating.

Despite these constraints, the ecological design remains a useful tool for identifying spatial patterns of risk and for informing the design of subsequent, more detailed studies [51].

### 4.6. Key Points

1. Between 2015 and 2024, mortality among children aged 6–17 years in Kazakhstan remained relatively low, but showed considerable variation across regions and calendar years, with no clear linear trend towards either increase or decrease.
2. Across the entire study period, the leading contributors to school-age mortality were Injury, poisoning and certain other consequences of external causes (ICD-10: S00–T98), followed by diseases of the nervous system, neoplasms, circulatory diseases and respiratory diseases; the overall cause pattern was stable over time and largely similar between regions.
3. Although mortality levels differed between regions, the contribution of geography was minimal (ICC < 1%), indicating weak regional heterogeneity. The highest mortality rates from external causes were observed in Ulytau, Zhetysu, Zhambyl, Pavlodar and Kostanay regions.
4. Poverty levels and gross regional product (GRP) per capita were low and relatively stable across all regions, whereas dietary indicators varied substantially. However, in multivariable models none of the socioeconomic or dietary predictors showed a statistically significant association with mortality among children aged 6–17 years (all *p* > 0.05).
5. Residual autocorrelation and heteroscedasticity pointed to the panel nature of the data and likely non-linear temporal dynamics, resulting in low explanatory power of the models (marginal R^2^ = 1.7%) and underscoring the limitations of simple linear mixed-effects specifications for this outcome.

## 5. Conclusions

Overall, the findings of this study indicate that mortality among school-aged children in Kazakhstan is shaped by a complex interplay of medical, social and behavioral determinants. Nutrition and socioeconomic status do influence mortality levels, but these effects are not uniform and need to be interpreted in the context of regional living conditions, access to healthcare and broader environmental factors. Adequate consumption of dairy and cereal products may act as a potential protective factor. Improving household incomes, strengthening social support and implementing coherent, balanced nutrition policies are likely to contribute to reducing mortality in the school-age population.

## Figures and Tables

**Table 1:** Median (IQR) number of children aged 6–17 years by region of Kazakhstan, 2015–2024.

**Region**	**Median Number of Children, n (IQR)**
Abai	102,290 (101,480–102,494)
Akmola	130,067 (120,304–137,949)
Aktobe	157,439 (137,217–175,159)
Almaty city	292,658 (253,952–331,436)
Almaty region	372,929 (355,152–414,560)
Astana city	188,720 (148,959–238,189)
Atyrau	133,984 (117,844–144,528)
East Kazakhstan	185,525 (101,805–192,794)
Karaganda	189,510 (175,974–203,293)
Kostanay	110,953 (105,897–114,715)
Kyzylorda	167,668 (149,293–180,626)
Mangystau	155,744 (129,906–175,795)
North Kazakhstan	74,122 (72,622–75,171)
Pavlodar	112,268 (100,942–116,206)
Shymkent city	226,545 (200,275–251,596)
Turkistan	506,532 (469,110–521,723)
Ulytau	40,628 (39,481–41,218)
West Kazakhstan	110,682 (100,198–119,580)
Zhambyl	237,568 (217,602–246,293)
Zhetysu	132,076 (130,965–133,353)

**Table 2:** Median age-specific mortality rates (per 100,000 children aged 6–17 years) by major ICD-10 cause categories, 2015–2024.

**ICD-10 Code**	**Me (Q1–Q3)**
XIX Injury, poisoning and certain other consequences of external causes (S00–T98)	11.29 [5.13–17.45]
VI Diseases of the nervous system (G00–G99)	4.39 [2.92–5.87]
II Neoplasms (C00–D48)	2.34 [1.26–3.42]
IX Diseases of the circulatory system (I00–I99)	1.63 [0.87–2.4]
X Diseases of the respiratory system (J00–J99)	1.53 [0.77–2.29]

**Table 3:** Median per capita consumption of major food products and gross regional product (GRP) by region of the Republic of Kazakhstan, 2015–2024.

**Region**	**Bread (kg per Capita per Year)**	**Meat (kg per Capita per Year)**	**Fish (kg per Capita per Year)**	**Milk (L per Capita per Year)**	**Eggs (Pieces per Capita per Year)**	**Oils (kg per Capita per Year)**	**Fruits (kg per Capita per Year)**	**Vegetables (kg per Capita per Year)**	**Potatoes (kg per Capita per Year)**	**Sugar (kg per Capita per Year)**	**GRP**
Abai	123.2 (120.6–124.3)	72.1 (66.3–72.6)	12.5 (11.9–12.6)	227.5 (216.5–244.3)	186.6 (169.3–191.5)	14.1 (13.3–14.3)	53.9 (50.2–55.7)	56.3 (54.2–57.8)	29.6 (28.7–30.7)	35.9 (32.3–39.9)	2,505,040 (2,246,826–2,781,901)
Akmola	135.0 (127.7–147.0)	92.0 (74.2–93.3)	17.3 (10.1–18.6)	285.1 (262.5–314.0)	248.7 (237.6–255.0)	20.5 (19.4–22.1)	69.7 (53.2–77.1)	68.4 (66.1–72.2)	51.0 (49.0–54.3)	46.0 (41.3–50.7)	2,108,760 (1,552,704–3,484,573)
Aktobe	128.0 (120.9–128.9)	72.6 (67.4–74.9)	11.7 (8.8–12.0)	226.3 (210.0–247.3)	164.4 (160.0–172.0)	16.5 (15.7–18.4)	73.2 (70.3–75.7)	65.5 (61.8–71.9)	46.0 (42.6–49.0)	42.8 (41.0–43.6)	2,965,647 (2,341,889–4,187,588)
Almaty city	103.8 (103.4–110.8)	84.9 (82.1–89.0)	14.5 (11.4–15.3)	265.1 (261.5–290.5)	221.2 (208.2–228.9)	17.9 (15.8–19.3)	90.8 (78.9–97.8)	97.6 (95.4–99.4)	44.7 (44.0–46.7)	44.3 (42.3–47.1)	13,503,381 (11,893,226–19,154,537)
Almaty region	148.4 (134.5–153.0)	87.9 (79.3–92.8)	13.6 (12.0–14.7)	226.0 (212.3–247.5)	190.1 (156.0–193.1)	14.0 (13.1–15.1)	71.6 (68.8–74.0)	90.6 (77.9–98.1)	42.4 (41.7–44.1)	45.9 (43.4–46.9)	3,312,907 (2,472,042–4,267,665)
Astana city	105.3 (102.6–106.7)	73.3 (70.8–80.7)	11.7 (8.0–12.4)	264.1 (250.4–272.1)	215.6 (186.6–228.0)	13.1 (11.7–15.5)	83.2 (69.3–89.9)	76.7 (72.8–81.5)	46.4 (45.2–46.9)	36.0 (34.6–37.1)	7,905,056 (5,775,621–10,672,481)
Atyrau	129.5 (126.2–136.5)	90.3 (87.2–93.6)	19.0 (18.4–19.0)	238.5 (192.4–242.2)	170.8 (161.4–175.0)	16.3 (14.6–20.8)	64.6 (58.9–66.9)	69.6 (66.8–78.2)	46.4 (41.3–48.5)	39.9 (36.2–46.4)	8,573,038 (5,947,654–13,725,400)
East Kazakhstan	138.5 (120.5–141.5)	87.8 (86.4–90.3)	19.2 (13.5–19.5)	289.1 (276.1–294.9)	217.2 (186.7–224.4)	16.2 (15.7–21.1)	73.2 (58.2–75.0)	73.1 (66.8–88.9)	49.1 (42.9–50.7)	41.8 (41.3–43.9)	3,753,075 (3,174,813–4,459,056)
Karaganda	125.5 (111.7–133.6)	86.5 (79.8–92.2)	14.2 (10.0–15.8)	275.3 (262.6–291.4)	247.8 (220.2–270.0)	22.4 (18.9–23.4)	83.0 (64.7–88.3)	80.6 (71.0–89.2)	59.9 (50.7–60.8)	47.2 (45.7–50.2)	5,728,809 (4,284,363–7,278,059)
Kostanay	119.3 (117.4–122.3)	76.2 (69.2–82.2)	17.6 (12.4–19.2)	210.2 (201.5–219.4)	240.9 (194.8–253.4)	17.3 (16.7–19.7)	64.0 (54.6–70.0)	79.5 (78.4–83.4)	52.1 (47.1–54.6)	40.8 (39.2–43.0)	2,661,973 (1,850,281–4,182,078)
Kyzylorda	142.0 (133.2–151.2)	63.8 (61.9–65.2)	16.0 (12.7–16.0)	189.0 (184.1–205.6)	146.6 (122.7–153.8)	17.9 (15.3–21.4)	76.4 (72.1–80.1)	84.6 (77.1–87.1)	42.7 (42.1–43.3)	42.5 (39.5–47.4)	1,737,941 (1,430,980–2,417,399)
Mangystau	107.0 (102.0–116.4)	84.1 (81.7–87.1)	9.6 (8.7–10.2)	229.2 (203.5–239.7)	128.5 (127.3–130.5)	18.1 (18.1–18.2)	85.6 (80.2–89.4)	81.7 (74.9–83.9)	51.2 (46.8–51.8)	38.3 (37.2–39.8)	3,656,196 (3,074,393–4,401,193)
North Kazakhstan	119.2 (111.7–121.5)	76.9 (69.8–80.1)	20.2 (15.6–21.2)	247.4 (240.1–248.3)	238.1 (216.3–252.4)	15.2 (14.5–18.1)	73.8 (57.9–76.4)	68.0 (60.6–74.7)	48.7 (47.3–50.5)	40.8 (37.8–42.5)	1,477,113 (1,113,959–2,198,854)
Pavlodar	128.4 (122.2–137.5)	87.5 (81.6–90.2)	13.9 (13.3–14.5)	281.8 (254.3–302.1)	187.2 (177.2–192.4)	16.7 (16.6–17.6)	69.5 (65.1–73.0)	84.9 (78.0–89.7)	53.9 (51.2–55.6)	42.3 (36.9–45.4)	3,074,873 (2,369,298–4,296,924)
Shymkent city	133.8 (127.6–157.1)	54.5 (53.1–58.2)	5.4 (4.9–8.8)	165.7 (155.7–208.0)	165.4 (163.2–182.0)	13.2 (12.9–16.5)	65.2 (60.3–66.6)	88.6 (82.9–106.9)	44.5 (38.7–48.1)	33.7 (32.5–37.7)	2,348,099 (1,712,054–3,294,392)
Turkistan	172.3 (164.9–173.3)	61.5 (58.2–62.5)	12.4 (8.9–12.5)	213.2 (208.6–228.9)	166.1 (149.0–166.6)	19.0 (17.2–21.3)	68.2 (66.6–70.8)	108.1 (101.7–110.0)	44.3 (43.3–45.6)	42.4 (41.9–43.3)	2,200,140 (1,475,670–3,517,281)
Ulytau	119.0 (113.5–120.5)	80.8 (77.1–82.1)	11.8 (10.8–11.9)	254.0 (247.7–255.1)	229.9 (215.8–237.9)	21.8 (20.3–22.9)	75.7 (66.6–75.9)	70.2 (63.2–72.2)	45.7 (42.9–47.5)	59.0 (52.6–60.6)	1,789,734 (1,551,534–2,099,195)
West Kazakhstan	136.0 (125.4–142.1)	79.2 (69.9–84.1)	15.3 (13.3–18.0)	226.5 (224.7–247.3)	165.7 (146.6–187.5)	19.2 (18.5–20.6)	69.2 (57.6–73.7)	78.0 (74.3–82.4)	54.6 (47.8–57.3)	38.0 (36.4–39.9)	2,868,525 (2,337,506–4,435,131)
Zhambyl	137.7 (126.6–141.6)	74.5 (68.3–84.1)	13.7 (12.1–14.6)	228.0 (199.1–236.8)	169.0 (126.0–180.6)	17.9 (16.8–20.0)	72.6 (58.8–76.0)	86.2 (81.5–90.5)	47.5 (46.2–48.1)	44.4 (40.1–45.9)	1,807,134 (1,350,662–2,685,460)
Zhetysu	141.7 (141.3–149.6)	93.4 (93.2–97.2)	14.0 (13.1–14.5)	244.1 (234.9–254.5)	155.0 (151.2–155.2)	13.7 (13.3–14.1)	74.0 (70.1–75.2)	85.5 (84.1–87.5)	39.3 (38.9–41.5)	48.1 (45.5–48.3)	1,618,150 (1,376,926–1,914,049)

**Table 4:** Median proportion of the population with incomes below the subsistence minimum by region of the Republic of Kazakhstan, 2015–2024.

Region	Poverty Level, Median (Q1–Q3), %
Abai	0.2 (0.1–0.6)
Akmola	0.2 (0.2–0.4)
Aktobe	0.1 (0.1–0.1)
Almaty city	0.1 (0.1–0.2)
Almaty region	0.1 (0.1–0.2)
Astana city	0.1 (0.1–0.1)
Atyrau	NA
East Kazakhstan	0.4 (0.2–0.4)
Karaganda	0.2 (0.1–0.2)
Kostanay	0.1 (0.1–0.2)
Kyzylorda	0.1 (0.1–0.1)
Mangystau	0.2 (0.1–0.4)
North Kazakhstan	0.2 (0.1–0.6)
Pavlodar	0.0 (0.0–0.0)
Shymkent city	0.3 (0.1–0.7)
Turkistan	0.2 (0.1–0.2)
Ulytau	0.6 (0.5–0.6)
West Kazakhstan	0.2 (0.1–0.3)
Zhambyl	0.1 (0.1–0.1)
Zhetysu	0.2 (0.1–0.8)

**Table 5:** Multivariable linear mixed-effects regression model for mortality rates among school-aged children (6–17 years).

**Predictor**	**β Estimate**	**95% CI**	***p*** **-Value**
Bread & cereal products	−0.017	−0.035 to +0.009	0.062
Meat and meat products	−0.024	−0.061 to +0.013	0.204
Fish and seafood	−0.022	−0.119 to +0.075	0.637
Milk and dairy products	−0.001	−0.014 to +0.011	0.891
Eggs	+0.002	−0.009 to +0.013	0.758
Oils and fats	−0.108	−0.226 to +0.010	0.072
Fruits	−0.021	−0.052 to +0.010	0.195
Vegetables	+0.000	−0.027 to +0.028	0.993
Potato	+0.018	−0.040 to +0.075	0.537
Sugar and confectionery	+0.031	−0.035 to +0.098	0.335
Poverty	+0.631	−0.576 to +1.838	0.305
Year	+0.094	−0.045 to +0.232	0.180

## Data Availability

Dataset available on request from the authors.

## Supplementary Materials

The following supporting information can be downloaded at: https://www.mdpi.com/article/10.3390/ijerph23010039/s1, Table S1. Top five causes of death among children aged 6–17 years by region of Kazakhstan, 2015–2024.
