# Supplementary material for: Nutrition and Social Disadvantage as Risk Factors for Mortality Among School-Age Children: Regional Differences in Kazakhstan
Source: Int J Environ Res Public Health. 2025 Dec 27;23(1):39. doi: 10.3390/ijerph23010039 (PMC12841260; doi:10.3390/ijerph23010039)
Supplement: Supplementary file 1 [file ijerph-23-00039-s001.zip › ijerph-4025634-supplementary.pdf]

Supplementary Table 1. Top five causes of death among children aged 6–17 years by region of Kazakhstan, 2015–2024

	Region	ICD-10	Mortality rate per 100,000, median (Q1–Q3)
**1**	Abai	XIX Injury, poisoning and certain other consequences of external causes (S 00-T98)	16.59 [15.19–20.51]
**2**	Abai	VI Diseases of the nervous system (G00-G99)	4.89 [4.88–4.91]
**3**	Abai	X Diseases of the respiratory system (J00-J99)	2.93 [2.44–2.94]
**4**	Abai	IX Diseases of the circulatory system (I00-I99)	2.44 [2.20–2.69]
**5**	Abai	XVIII Symptoms, signs and abnormal clinical and laboratory findings, not elsewhere classified (R00-R99)	1.97 [1.97–1.97]
**6**	Akmola	XIX Injury, poisoning and certain other consequences of external causes (S 00-T98)	10.11 [7.24–11.11]
**7**	Akmola	VI Diseases of the nervous system (G00-G99)	3.23 [1.88–5.33]
**8**	Akmola	X Diseases of the respiratory system (J00-J99)	2.27 [1.58–2.58]
**9**	Akmola	IX Diseases of the circulatory system (I00-I99)	1.78 [1.51–2.42]
**10**	Akmola	II Neoplasms (C00-D48)	1.59 [0.81–2.30]
**11**	Aktobe	XIX Injury, poisoning and certain other consequences of external causes (S 00-T98)	12.14 [6.29–16.33]

**12**	Aktobe	VI Diseases of the nervous system (G00-G99)	4.90 [4.36–5.41]
**13**	Aktobe	II Neoplasms (C00-D48)	2.48 [1.11–3.04]
**14**	Aktobe	X Diseases of the respiratory system (J00-J99)	2.28 [1.34–3.05]
**15**	Aktobe	IX Diseases of the circulatory system (I00-I99)	1.51 [1.09–1.93]
**16**	Almaty city	XIX Injury, poisoning and certain other consequences of external causes (S 00-T98)	15.27 [6.68–23.68]
**17**	Almaty city	II Neoplasms (C00-D48)	8.07 [6.94–8.72]
**18**	Almaty city	VI Diseases of the nervous system (G00-G99)	3.36 [2.76–4.90]
**19**	Almaty city	X Diseases of the respiratory system (J00-J99)	1.71 [1.20–2.36]
**20**	Almaty city	IX Diseases of the circulatory system (I00-I99)	1.58 [1.34–1.94]
**21**	Almaty regi on	XIX Injury, poisoning and certain other consequences of external causes (S 00-T98)	4.18 [2.61–6.25]
**22**	Almaty regi on	VI Diseases of the nervous system (G00-G99)	3.75 [3.30–4.23]
**23**	Almaty regi on	II Neoplasms (C00-D48)	1.49 [0.88–2.05]
**24**	Almaty regi on	IX Diseases of the circulatory system (I00-I99)	0.95 [0.59–1.54]

**25**	Almaty regi on	X Diseases of the respiratory system (J00-J99)	0.95 [0.48–1.76]
**26**	Astana city	II Neoplasms (C00-D48)	10.01 [9.34–11.42]
**27**	Astana city	XIX Injury, poisoning and certain other consequences of external causes (S 00-T98)	7.39 [1.51–11.01]
**28**	Astana city	VI Diseases of the nervous system (G00-G99)	3.49 [3.03–4.05]
**29**	Astana city	X Diseases of the respiratory system (J00-J99)	2.85 [0.69–3.34]
**30**	Astana city	IX Diseases of the circulatory system (I00-I99)	1.79 [1.38–2.29]
**31**	Atyrau	VI Diseases of the nervous system (G00-G99)	5.17 [3.62–6.54]
**32**	Atyrau	XIX Injury, poisoning and certain other consequences of external causes (S 00-T98)	2.93 [0.90–14.95]
**33**	Atyrau	XX External causes of morbidity and mortality (V01-Y98)	2.85 [2.40–3.61]
**34**	Atyrau	II Neoplasms (C00-D48)	2.58 [1.50–3.57]
**35**	Atyrau	XVII Congenital malformations, deformations and chromosomal abnormali ties (Q00-Q99)	2.09 [1.62–2.38]
**36**	East Kazakh stan	XIX Injury, poisoning and certain other consequences of external causes (S 00-T98)	12.46 [10.53–14.56]
**37**	East Kazakh stan	VI Diseases of the nervous system (G00-G99)	1.84 [1.57–2.89]

**38**	East Kazakh stan	X Diseases of the respiratory system (J00-J99)	1.73 [0.76–1.98]
**39**	East Kazakh stan	IX Diseases of the circulatory system (I00-I99)	1.50 [0.77–1.97]
**40**	East Kazakh stan	I Certain infectious and parasitic diseases	1.48 [0.65–3.49]
**41**	Karaganda	XIX Injury, poisoning and certain other consequences of external causes (S 00-T98)	4.89 [1.68–15.91]
**42**	Karaganda	VI Diseases of the nervous system (G00-G99)	2.62 [2.15–4.08]
**43**	Karaganda	II Neoplasms (C00-D48)	1.62 [0.69–2.26]
**44**	Karaganda	I Certain infectious and parasitic diseases	1.35 [1.07–1.97]
**45**	Karaganda	X Diseases of the respiratory system (J00-J99)	1.14 [1.00–1.61]
**46**	Kostanay	XIX Injury, poisoning and certain other consequences of external causes (S 00-T98)	16.67 [15.23–20.47]
**47**	Kostanay	X Diseases of the respiratory system (J00-J99)	3.62 [2.86–5.09]
**48**	Kostanay	VI Diseases of the nervous system (G00-G99)	2.27 [1.77–2.77]
**49**	Kostanay	IX Diseases of the circulatory system (I00-I99)	2.19 [1.56–2.65]
**50**	Kostanay	XX External causes of morbidity and mortality (V01-Y98)	1.86 [1.86–1.86]

**51**	Kyzylorda	VI Diseases of the nervous system (G00-G99)	3.91 [3.38–4.34]
**52**	Kyzylorda	XIX Injury, poisoning and certain other consequences of external causes (S 00-T98)	3.87 [2.39–12.00]
**53**	Kyzylorda	I Certain infectious and parasitic diseases	2.75 [1.28–3.93]
**54**	Kyzylorda	II Neoplasms (C00-D48)	2.53 [1.53–3.28]
**55**	Kyzylorda	XVIII Symptoms, signs and abnormal clinical and laboratory findings, not elsewhere classified (R00-R99)	1.31 [0.56–1.46]
**56**	Mangystau	VI Diseases of the nervous system (G00-G99)	5.54 [4.02–6.47]
**57**	Mangystau	XIX Injury, poisoning and certain other consequences of external causes (S 00-T98)	4.09 [1.73–10.24]
**58**	Mangystau	X Diseases of the respiratory system (J00-J99)	1.90 [1.39–2.52]
**59**	Mangystau	II Neoplasms (C00-D48)	1.83 [1.61–2.24]
**60**	Mangystau	XVIII Symptoms, signs and abnormal clinical and laboratory findings, not elsewhere classified (R00-R99)	1.63 [1.33–1.89]
**61**	North Kazak hstan	XIX Injury, poisoning and certain other consequences of external causes (S 00-T98)	13.36 [12.62–19.46]
**62**	North Kazak hstan	VI Diseases of the nervous system (G00-G99)	6.64 [5.39–7.65]

**63**	North Kazak hstan	XVII Congenital malformations, deformations and chromosomal abnormali ties (Q00-Q99)	2.74 [2.33–3.08]
**64**	North Kazak hstan	X Diseases of the respiratory system (J00-J99)	2.67 [1.36–2.75]
**65**	North Kazak hstan	IX Diseases of the circulatory system (I00-I99)	2.66 [2.01–3.37]
**66**	Pavlodar	XIX Injury, poisoning and certain other consequences of external causes (S 00-T98)	16.53 [13.30–17.34]
**67**	Pavlodar	VI Diseases of the nervous system (G00-G99)	4.79 [3.57–5.77]
**68**	Pavlodar	IV Endocrine, nutritional and metabolic diseases (E00-E90)	2.68 [2.22–3.14]
**69**	Pavlodar	II Neoplasms (C00-D48)	1.98 [1.69–3.14]
**70**	Pavlodar	XVIII Symptoms, signs and abnormal clinical and laboratory findings, not elsewhere classified (R00-R99)	1.69 [1.01–1.73]
**71**	Shymkent ci ty	XIX Injury, poisoning and certain other consequences of external causes (S 00-T98)	14.48 [11.32–39.75]
**72**	Shymkent ci ty	VI Diseases of the nervous system (G00-G99)	5.54 [4.80–7.28]
**73**	Shymkent ci ty	XX External causes of morbidity and mortality (V01-Y98)	3.97 [2.26–5.49]
**74**	Shymkent ci ty	IX Diseases of the circulatory system (I00-I99)	3.00 [1.85–3.73]

**75**	Shymkent city	I Certain infectious and parasitic diseases	2.66 [2.21–2.92]
**76**	Turkistan/SK	VI Diseases of the nervous system (G00-G99)	6.29 [3.81–7.79]
**77**	Turkistan/SK	XIX Injury, poisoning and certain other consequences of external causes (S00-T98)	4.79 [3.72–5.39]
**78**	Turkistan/SK	II Neoplasms (C00-D48)	2.78 [2.42–2.93]
**79**	Turkistan/SK	IX Diseases of the circulatory system (I00-I99)	1.23 [0.79–1.60]
**80**	Turkistan/SK	I Certain infectious and parasitic diseases	0.78 [0.37–1.50]
**81**	Ulytau	XIX Injury, poisoning and certain other consequences of external causes (S00-T98)	21.84 [21.05–24.46]
**82**	Ulytau	VI Diseases of the nervous system (G00-G99)	7.38 [6.12–13.82]
**83**	Ulytau	IX Diseases of the circulatory system (I00-I99)	3.67 [3.05–4.30]
**84**	Ulytau	XVII Congenital malformations, deformations and chromosomal abnormalities (Q00-Q99)	2.53 [2.53–2.53]
**85**	Ulytau	XI Diseases of the digestive system (K00-K93)	2.48 [2.45–2.51]
**86**	West Kazakhstan	XX External causes of morbidity and mortality (V01-Y98)	16.42 [14.34–21.08]

**87**	West Kazakhstan	VI Diseases of the nervous system (G00-G99)	6.01 [4.31–7.94]
**88**	West Kazakhstan	XIX Injury, poisoning and certain other consequences of external causes (S00-T98)	4.36 [2.46–11.17]
**89**	West Kazakhstan	II Neoplasms (C00-D48)	2.76 [1.67–3.17]
**90**	West Kazakhstan	IX Diseases of the circulatory system (I00-I99)	1.73 [1.24–2.07]
**91**	Zhambyl	XIX Injury, poisoning and certain other consequences of external causes (S00-T98)	16.65 [12.63–20.34]
**92**	Zhambyl	VI Diseases of the nervous system (G00-G99)	6.95 [5.46–8.43]
**93**	Zhambyl	II Neoplasms (C00-D48)	3.03 [1.35–3.63]
**94**	Zhambyl	IX Diseases of the circulatory system (I00-I99)	1.62 [0.93–2.12]
**95**	Zhambyl	XVII Congenital malformations, deformations and chromosomal abnormalities (Q00-Q99)	1.62 [1.30–1.62]
**96**	Zhetysu	XIX Injury, poisoning and certain other consequences of external causes (S00-T98)	21.20 [21.10–21.67]
**97**	Zhetysu	VI Diseases of the nervous system (G00-G99)	5.25 [4.90–7.97]
**98**	Zhetysu	IX Diseases of the circulatory system (I00-I99)	3.00 [2.65–3.39]

**99**	Zhetysu	II Neoplasms (C00-D48)	1.50 [1.50–1.50]
**100**	Zhetysu	IV Endocrine, nutritional and metabolic diseases (E00-E90)	1.50 [1.50–1.50]

Values are cause-specific mortality rates per 100,000 children aged 6–17 years. Results are presented as median (Q1–Q3) across 2015–2024; Q1 and Q3 denote the 25th and 75th percentiles, respectively.
